# Measures of empathy and the capacity for self-reflection in dental and medical students

**DOI:** 10.1186/s12909-021-02549-3

**Published:** 2021-02-18

**Authors:** Elina Paloniemi, Ilona Mikkola, Ritva Vatjus, Jari Jokelainen, Markku Timonen, Maria Hagnäs

**Affiliations:** 1 Center for Life Course Health Research, University of Oulu, Oulu, Finland; 2 Rovaniemi Health Center, Rovaniemi, Finland

**Keywords:** Medical education, Empathy, Self-reflection

## Abstract

**Background:**

Empathy and self-reflection have been studied among medical students, but fewer studies have examined the presence of these attributes among dental students and investigated the correlation between empathy and self-reflection.

**Methods:**

First-year dental and medical students (*n* = 198) beginning their studies at the University of Oulu, Finland in August 2017 participated in this study, which was conducted via an internet-based questionnaire. Data were collected on personal characteristics and scores on Davis’s Interpersonal Reactivity Index (IRI) and Roberts’s Self Reflection and Insight Scale (SRIS).

**Results:**

Differences in IRI scores between dental and medical students were significant only in male students and in two IRI domains. Mean (SD) scores for male dental and medical students were personal distress, 8.2 (4.0) and 10.7 (3.1) (*p* = 0.022); empathic concern, 15.0 (4.0) and 16.9 (3.5) (*p* = 0.054). Mean SRIS scores did not differ between sexes or training programs. Positive correlations (*r* = − 0.3–0.65) were observed between some empathy and self-reflection subscales.

**Conclusions:**

A lower degree of empathy was observed among male dental students than in male medical students. A positive correlation between empathy and self-reflection was demonstrated in both study groups and sexes. However, more research in this field is warranted.

## Background

With regard to medical education, empathy has recently been defined as the ability to understand patient’s situation, perspective and feelings, and to communicate that understanding to the patient, and includes cognitive, emotional and behavioral elements [1, 2]. Furthermore, Sulzer et al. (2016) suggest approaching empathy as a relational subject rather than a personal quality [2]. Self-reflection in medical education can be defined as critical and conscious thought about one’s behavior and practice [3] or more profoundly as careful exploration and evaluation of experiences [4]. In recent years research into empathy and self-reflection in medical education has intensified**.**

There is evidence that self-reflection and reflective practice can be taught and developed during basic medical education through mentorship, supervision and peer support [5]. Furthermore, the process of reflection and capacity to conduct reflective practise enhances learning during medical education [6] and may improve self-understanding [5]. Because of empathy’s cognitive nature it leads to personal growth and is salutary to the physician-patient relationship, making it a worthwhile component of medical education [7]. Even though self-reflection has been suggested to be a prerequisite for the development of empathy [8], there is a lack of research in this field [9].

Empathy levels in medical students are reported to differ according to sex, with females tending to score higher than males [10–12]. However, there is a lack of data on empathy and self-reflection among students of dentistry. To the best of our knowledge, no previous studies have examined the possible difference in empathy and self-reflection ability between dental and medical students. The primary aim of this study was to measure the mean empathy and self-reflection scores among medical and dental students. Our secondary aim was to evaluate the correlation between empathy and self-reflection questionnaire scores. Our hypothesis was that mean empathy and self-reflection scores would not differ between medical and dental students.

## Methods

### Participants

**Fig. 1:**
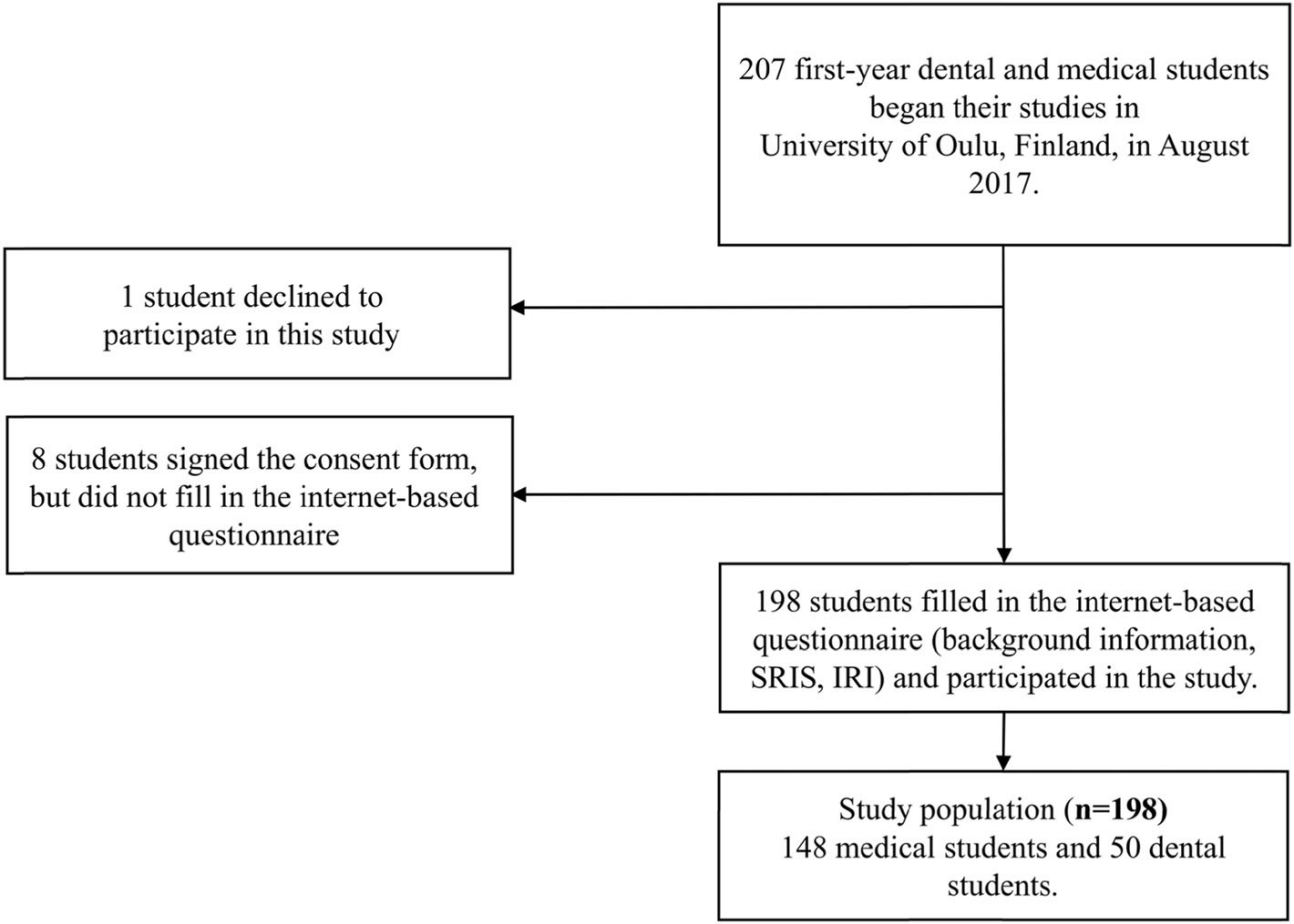
Formation of study population

All first-year dental and medical students who began their studies at the University of Oulu, Finland in August 2017 were invited to participate in the study, which was conducted in the Fall semester of 2017. The students were informed about this voluntary study as a part of compulsory lecture of general practice course, which is a part of the medical curriculum. Medical education in Finnish universities consists of a six-year program, of which first 2 years are preclinical studies. Dental education takes a total of 5 years, with the first 2 years being mainly the same as those of the medical program. During compulsory group session, students were given the opportunity to sign the informed consent form and fill in the internet-based questionnaire, but participation in the study was not mandatory. Of all 207 first-year students, 206 gave consent for use of the collected data for scientific purposes and, of those, 202 also gave permission for the use of data regarding their entrance exam results. Finally, 198 students filled in the internet-based questionnaire and participated in the study. Of those, 148 were medical students and 50 dental students. Formation of study population is shown in Fig. 1. The study protocol was approved by the Ethics Committee of the Northern Ostrobotnia Hospital District, Finland. All methods were performed in accordance with the relevant guidelines and regulations.

### Instruments

At baseline, students were asked to fill an internet-based questionnaire, which gathered information about their age, previous studies and/or profession, participation in training courses before the entrance exam, their number of attempts at the medical school entrance exam and their parents’ professions.

The internet-based study questionnaire also evaluated the participants’ levels of empathy and self-reflection ability, using the Interpersonal Reactivity Index (IRI) [13] and the Self Reflection and Insight Scale (SRIS) [14]. The IRI was developed to evaluate an individual’s level of empathy. It includes 28 items, each answered on a 5-point Likert scale from “Does not describe me well” (zero points) to “Describes me very well” (four points), with negatively-worded items scored in reverse. The items are divided into four subscales, each with 7 items: perspective taking (PT; ability to adopt another person’s psychological point of view), personal distress (PD; propensity to react with feelings of personal distress in response to another person’s distress) empathic concern (EC; tendency to feel concern for other people) and fantasy (F; the tendency to become imaginatively involved with fictional characters and situations) [13]. The SRIS was developed to evaluate an individual’s capacity for self-reflection. It includes 20 items also answered on a 5-point Likert scale. In that case statements are scored from one to five with one equating to “Strongly agree” and five to “Strongly disagree” except for reversed-scored items, which are scored from five to one. The statements are related to three levels of reflection: need for self-reflection (NSR), engaging in self-reflection (ESR) and insight (I). The maximum possible score is 30 points for both the “NSR” and “ESR” components, and 40 points for the “I” dimension [14].

### Statistical analysis

Data are presented as mean, standard deviation (SD) and 95% CI (confidence interval). The independent samples t-test was used to compare the mean values between groups. Statistical analyses were performed using IBM SPSS Statistics for Macintosh, Version 24.0. Armonk, NY: IBM Corp. IBM Corp. Released 2016. A *p*-value < 0.05 was considered statistically significant. Spearman correlation coefficients were used to assess associations between the Interpersonal Reactivity Index (IRI) and the Self Reflection and Insight Scale (SRIS). Heatmaps were used to represent the correlations of these two scales. Correlation coefficients were used to categorise effect size as follows: small (0.1–0.3), medium (0.3–0.5) and large (0.5–1.0) [15]. The Fisher’s r-to-z approach was used to compare correlation coefficients [16].

## Results

Evaluable data were available for 198 participants, of whom 87 (42.0%) were male and 148 (74.7%) were medical students. The mean age was 22.6 years (SD 4.1, range 19.0–42.0). A total of 150 participants (72.5%) had attended at least one training course before their entrance exam and the mean (SD) number of attempts at the entrance exam before acceptance to the Medical Faculty was 2.4 (1.1). Between the matriculation exam and entry to the Medical Faculty, 57 participants (28.8%) had a profession or undertook other studies; 2.9% had completed a master’s degree and 7.2% had unfinished education. There were no differences in baseline information between students according to sex or training program.

Mean scores on the IRI and SRIS subscales are shown in Table 1 (stratified according to sex) and in Table 2 (stratified according to training program). Mean scores for three of the four IRI subscales, fantasy, empathic concern and personal distress, were significantly higher in female students than in males (*p* ≤ 0.013 for each). Overall, there were no study-program-dependent differences in mean scores on any subscale, but when male students were analysed separately, male medical students had a higher mean (SD) score (10.7 [3.1]) for the IRI personal distress scale than male dental students (8.2 [4.0]; *p* = 0.022). No such difference was seen among female students. Neither sex nor training program resulted in any significant difference in SRIS scores. Mean (SD) score for the SRIS engagement in reflection scale was 20.5 (3.8) in female dental students and 19.6 (3.4) in male dental students. These values were 19.6 (4.2) and 19.2 (4.7) in female and male medical students, respectively. Although the mean scores on this subscale were higher in female students, the sex differences did not reach significance.

**Table 1:** Descriptive statistics for the components of the Self Reflection and Insight scale (SRIS) and Interpersonal Reactivity Index (IRI) according to sex

	All students (*n* = 198)		Female students (*n* = 111)		Male students (*n* = 87)		
	Mean (SD)	95% CI	Mean (SD)	95% CI	Mean (SD)	95% CI	p
**SRIS**							
Engage in reflection (/30)	19.6 (4.5)	19.0–20.2	19.9 (4.4)	19.1–20.7	19.3 (4.5)	18.3–20.2	0.318
Need for reflection (/30)	22.5 (4.3)	21.9–23.1	22.7 (4.3)	21.9–23.5	22.2 (4.4)	21.2–23.1	0.399
Insight (/40)	24.1 (4.0)	23.6–24.7	23.8 (4.1)	23.0–24.6	24.6 (3.7)	23.8–25.3	0.193
**IRI**							
Perspective-taking scale (/28)	18.6 (4.2)	18.0–19.2	18.5 (4.5)	17.7–19.4	18.6 (3.8)	17.6–19.4	0.958
Fantasy scale (/28)	15.2 (5.5)	14.4–16.0	16.3 (5.6)	15.2–17.3	13.9 (5.2)	12.8–15.0	0.003
Empathic concern scale (/28)	17.4 (4.2)	16.8–18.0	18.9 (4.5)	17.2–18.9	16.5 (3.6)	15.7–17.3	0.013
Personal distress scale (/28)	9.5 (4.2)	9.0–10.1	10.2 (4.3)	9.4–11.0	8.6 (4.0)	7.8–9.5	0.009

SRIS and IRI maximum scores in parenthesis. P from independent samples t-test, difference between genders

**Table 2:** Descriptive statistics for the components of the Self Reflection and Insight Scale (SRIS) and Interpersonal Reactivity Index (IRI) according to study program

	All (*n* = 198)		Medical students (*n* = 148)		Dental students (*n* = 50)		
	Mean (SD)	95% CI	Mean (SD)	95% CI	Mean (SD)	95% CI	p
**SRIS**							
Engage in reflection (/30)	19.6 (4.5)	19.0–20.2	19.4 (4.4)	18.7–20.1	20.2 (4.5)	18.9–21.5	0.281
Need for reflection (/30)	22.5 (4.3)	21.9–23.1	22.5 (4.3)	21.8–23.2	22.3 (4.3)	21.0–23.5	0.726
Insight (/40)	24.1 (4.0)	23.6–24.7	24.2 (4.2)	23.6–24.9	23.8 (3.4)	22.9–24.8	0.543
**IRI**							
Perspective-taking scale (/28)	18.6 (4.2)	18.0–19.2	18.9 (4.1)	18.2–19.5	17.9 (4.3)	16.6–19.0	0.118
Fantasy scale (/28)	15.2 (5.5)	14.4–16.0	14.9 (5.8)	14.0–15.9	16.0 (5.1)	14.6–17.5	0.229
Empathic concern scale (/28)	17.4 (4.2)	16.8–18.0	17.2 (4.1)	16.6–17.9	17.7 (4.6)	16.4–19.0	0.501
Personal distress scale (/28)	9.5 (4.2)	9.0–10.1	9.2 (4.4)	8.5–9.9	10.5 (3.5)	9.5–11.5	0.048

SRIS and IRI maximum scores in parenthesis. P from independent samples t-test, difference between medical and dentistry students

**Fig. 2:**
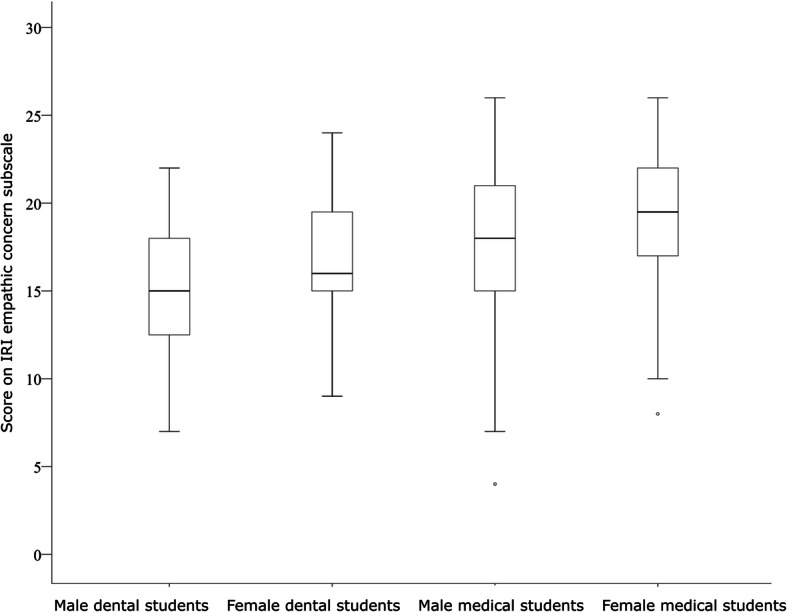
Scores on the empathic concern subscale (mean (SD)) of the Interpersonal Reactivity Index (IRI) in subjects stratified by sex and study program

Mean (SD) score on the IRI empathic concern scale was 15.0 (4.0) in male dental students, lower than the 16.9 (3.5) found in male medical students (*p* = 0.054). Neither sex nor training program explains this difference. The mean (SD) score on the IRI empathic concern scale was highest in female dental students: 19.0 (4.3). Figure 2 demonstrates the differences.

**Fig. 3:**
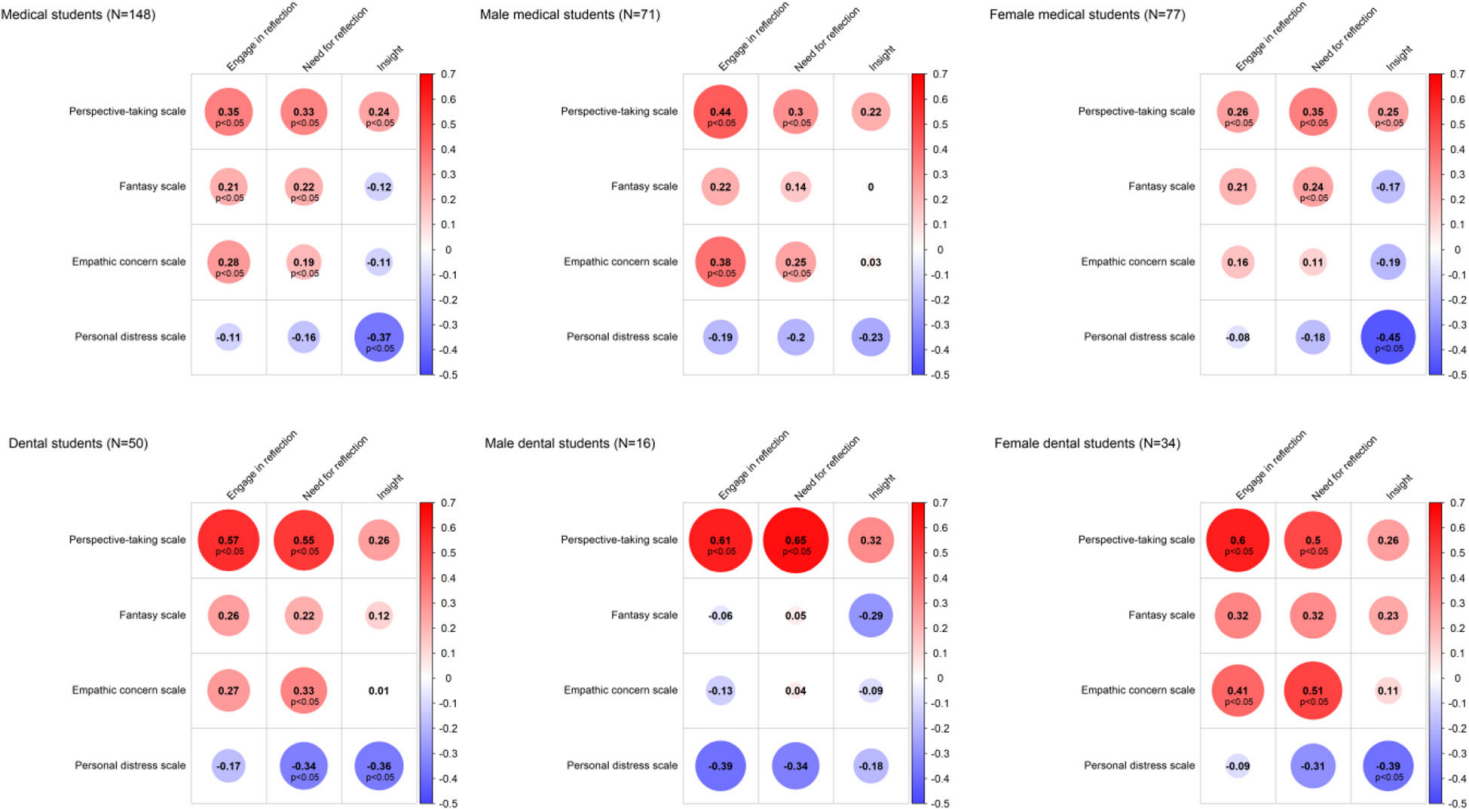
Spearman correlation heat maps for interpersonal reactivity index (IRI) and self-reflection insight scale (SRIS) according to sex and study program. Red represents a positive and blue represents a negative correlation. The darker and the more saturated color corresponds the greater magnitude of the correlation

Figure 3 shows the correlations between scores on the IRI and SRIS scales in male/female medical/dental students. Correlations of at least medium strength between the following dimensions were observed in both medical and dental students: IRI - PT and SRIS - ESR; IRI - PT and SRIS - NSR; IRI - PD and SRIS - I. Additionally, among dental students medium strength correlations were observed between SRIS - NSR and both EC and PD on the IRI scale. The correlations between EC and NSR; PT and ESR were significantly stronger in female dental students than in female medical students. The correlation between EC and ESR was positive among male medical students, but negative among male dental students. However, the difference was non-significant.

## Discussion

The present study was the first to evaluate the levels of empathy and the capacity for self-reflection among dental and medical students. We found that male dental students scored lower than male medical students in two of four IRI scales. Another of our main findings was that empathy and self-reflection correlated positively among all students.

Average scores for empathic concern and personal distress were lower in male dental students than in male medical students. One possible explanation for this could be that male students applying for the dental training program may perceive dental patient care as being more operational, thus placing less emphasis on interpersonal skills. However, this hypothesis is highly speculative and further research in this field is needed.

The previous literature concerning the correlation between empathy and self-reflection is scant [9], and to the best of our knowledge no previous study has examined the correlation between empathy and self-reflection scores in the context of medical education. Our finding of a positive correlation between empathy and self-reflection aligns with those of an earlier study in a population of college students, which found that enhanced self-reflection is positively correlated with perspective-taking and empathic concern [17]. Future research is needed to clarify the correlation.

Scores for empathy scales were higher in female students than in males, apart from for the perspective taking scale. Several earlier studies that used the IRI to evaluate levels of empathy in medical students similarly reported lower scores in males for the empathic concern [10, 11, 18, 19], personal distress [18, 19] and fantasy [19] subscales. In contrast with our own findings, several studies have also reported sex differences in scores for the perspective taking scale [10, 11, 18, 19]. The capacity for self-reflection did not differ statistically significantly between sexes, which is in line with previous studies [20–22].

We found no previous studies examining affective aspect of empathy and personal distress among dental students. Therefore, we believe that our novel findings are important given that empathy has a crucial role in dental patient care: in a review article by Jones et al. a high level of empathy among dentists was associated with the implementation of negotiated treatment plans, greater treatment adherence, increased patient satisfaction, and reduced patient anxiety [23].

We chose the IRI to measure levels of empathy. Several instruments have been used previously to measure medical students’ levels of empathy and capacity for self-reflection [13, 14, 24–29] the most widely used being The Jefferson Scale of Empathy for Students (JSE-S) and the IRI. Findings using the JSE-S and IRI appear to correlate only weakly, which suggests that they may measure different constructs of empathy [30]. Therefore, our results cannot be compared directly with those of studies that used the JSE-S. However, in our opinion, comparisons may be made at a general level, concerning the measured differences between sexes and training programs. To measure the capacity for self-reflection, we chose the SRIS, which was developed and validated specifically for medical students [14].

A high degree of participation on the part of our selected population (99%) can be considered a major strength of this study. Our population provides a representative sample of first-year students in the Medical Faculty of the University of Oulu. Although the study questionnaire was administered during a compulsory group session, participation was voluntary, and every student willing to attend was given a peaceful moment to complete the questionnaire. It was therefore possible for the supervising teacher to assist any student who required help with technical issues or unclear assignments. All participants answered all the questions and there was no need to exclude any of them from the analysis because of missing data. The ongoing follow-up study will provide data yearly until our group of students graduate.

## Conclusions

In conclusion, we report for the first time the levels of empathy and the capacity for self- reflection among dental and medical students in an ongoing prospective study. We observed a lower degree of empathy among male dental than male medical students. A positive correlation between empathy and self-reflection was demonstrated in both study groups and sexes. However, more research in this field is warranted. Active research, including qualitative analysis, is needed to confirm the role of empathy and self-reflection in dental and medical education.

## Data Availability

The dataset supporting the conclusions of this article is included within the article.

## Abbreviations

IRI: Interpersonal Reactivity Index
SRIS: Self Reflection and Insight Scale
